# Intra- and Inter-Specific Crosses among *Centaurea aspera* L. (Asteraceae) Polyploid Relatives—Influences on Distribution and Polyploid Establishment

**DOI:** 10.3390/plants9091142

**Published:** 2020-09-03

**Authors:** Alfonso Garmendia, María Ferriol, David Benavent, P. Pablo Ferrer-Gallego, Hugo Merle

**Affiliations:** 1 Instituto Agroforestal Mediterráneo, Universitat Politècnica de València, ES-46022 Valencia, Spain; algarsal@upvnet.upv.es (A.G.); mafermo@upvnet.upv.es (M.F.); 2 Departamento de Ecosistemas Agroforestales, Universitat Politècnica de València, ES-46022 Valencia, Spain; dabema@etsiamn.upv.es; 3 Servicio de Vida Silvestre, Centro para la Investigación y la Experimentación Forestal - VAERSA, Generalitat Valenciana, Avda. Comarques del País Valencià 114, Quart de Poblet, ES-46930 Valencia, Spain; flora.cief@gva.es

**Keywords:** allopolyploid, autopolyploid, cytotype, tetraploid, triploid, polyploidy, minority-cytotype exclusion, postzygotic barriers, *Centaurea*, Asteraceae

## Abstract

How polyploids become established is a long-debated question, especially for autopolyploids that seem to have no evolutionary advantage over their progenitors. The *Centaurea aspera* polyploid complex includes diploid *C. aspera* and two related tetraploids *C. seridis* and *C. gentilii*. Our purpose was to study the mating system among these three taxa and to analyze its influence on polyploid establishment. The distribution and ploidy level of the Moroccan populations, and forced intra- and inter-specific crosses were assessed. Allotetraploid *C. seridis* produced more cypselae per capitulum in the intra-specific crosses. It is a bigger plant and autogamous, and previous studies indicated that selfing forces the asymmetric formation of sterile hybrids. All these characteristics help *C. seridis* to avoid the minority-cytotype-exclusion effect and become established. Inter-specific hybridization was possible between *C. aspera* and *C. gentilii*, and with the symmetric formation of hybrids. However, 49% of the hybrid cypselae were empty, which probably reveals postzygotic barriers. Autotetraploid *C. gentilii* produced the same number of cypselae per capitulum as those of the diploid parental, has an indistinguishable field phenotype, is allogamous, and symmetrically produces hybrids. Therefore, *C. gentilii* does not seem to have the same competitive advantages as those of *C. seridis*.

## 1. Introduction

Polyploidy is one of the most important and ubiquitous driving forces in plant evolution [1,2,3]. In a climate-change context, Levin (2019) proposed that polyploidization will be one of the most frequent speciation modes in the next 500 years, with an increase in the percentage of polyploid plants from 35% today to more than 50%. This hypothesis is related to the fact that polyploidy can occur in sympatry, and neopolyploids can be established in only a few generations; therefore, they might be reinforced in rapidly changing scenarios [4].

Autopolyploids are considered to form by genome multiplication, while allopolyploids derive from hybridization between species with differentiated genomes, either by chromosome doubling after the fusion of reduced or unreduced gametes. However, this classification has long since been discussed. The term “segmental allopolyploid” is used to describe polyploids that do not exhibit strict bivalent formation across all chromosomes or disomic inheritance at all loci [5]. While autopolyploids and allopolyploids are identified according to chromosome pairing behavior (formation of multivalents and bivalents in meiosis, respectively), Stebbins (1947) considered that the parents of segmental allopolyploids occupied an intermediate level of chromosomal divergence between those of autopolyploids and allopolyploids [6].

How polyploids establish remains a debated question in evolutionary biology [7], especially autopolyploids that seem to have no clear evolutionary advantage over their diploid progenitors [8,9]. As a result, for years, autopolyploids were considered rare in nature, representing evolutionary dead ends [10,11]. However, recent studies showed that autopolyploids are more abundant than expected [12,13], and their abundance could have been underestimated due to recognition difficulties, as their phenotypes are similar or identical to their diploid progenitors [14].

To analyze the evolutionary significance of polyploidy, Levy and Feldman (2004) differentiated short-term “revolutionary changes”, related mainly to the establishment of polyploids, and long-term “evolutionary changes”, related more to their expansion and persistence. In this sense, allopolyploids undergo extensive genomic changes in first generations [15,16,17], while autopolyploids may experience fewer structural changes [13,18]. Unlike nascent autopolyploids, well-established autopolyploids can also show substantial genome reorganization compared to their diploid relatives [8,13,19]. Along with these genomic changes, functional reorganization of the gene-expression network may also occur, which is much more evident in allopolyploids than in autopolyploids [20,21,22]. Recent studies considered autopolyploidy as a macromutation with epigenetic consequences [23]. In general, hybridization (included in allopolyploid formation) seems to trigger significant changes, while only genome doubling maintains a similar state to that of its diploid progenitor [6,8,24]. In addition, autopolyploids have always been expected to be less fertile than allopolyploids are given the meiotic irregularities caused by multivalent chromosome pairing [25]. However, more recent studies revealed that aberrant meiosis affects both auto- and allopolyploids [17,26]. Such irregularities may be overcome through the frequent turnover of reproduction modes (from sexual to apomictic reproduction) [27,28] or the evolution of a stabilized form of meiotic asymmetry in chromosome inheritance [29].

The union of unreduced (2n) gametes is thought to be the commonest pathway for natural polyploid formation [30] through either the fusion of two unreduced gametes or a “triploid bridge” that can generate tetraploid progeny through selfing or backcrossing [31]. Triploids are often sterile [32,33], but they can sometimes produce a large proportion of fertile unreduced gametes that increases the possibility of tetraploid formation [31,34]. The production of unreduced gametes has been proven heritable, governed by a few genes, and increasing with environmental stress, such as heat, frost, water deficit, and herbivory [8,35,36]. This is in accordance with the higher frequency of polyploids found in habitats affected by climate fluctuations and disruptions [37,38,39]. Some studies pointed out that polyploid species are over-represented in previously glaciated regions, while diploids are more frequent in disjunct refugial areas [40,41]. Under these abiotic conditions, polyploid formation can be recurrent and with multiple origins. Despite all this knowledge, the establishment of neopolyploids and especially neo-auto-polyploids remains unclear.

*Centaurea* (Asteraceae) is a taxonomically intricate genus due to the existence of polyploidy, descending dysploidy cycles, and hybridization events, with a large number of polyploid complexes [42,43]. The *Centaurea aspera* L. polyploid complex has long since been studied, and it is mainly distributed in coastal habitats of Spain and Morocco. [32,38,44,45,46,47,48,49,50,51]. What makes the *C. aspera* polyploid complex so interesting is that it is made up of natural populations of the parental diploid (*C. aspera* L. 2x = 22), an allotetraploid (*C. seridis* L. 4x = 44) and an autotetraploid (*C. gentilii* Braun-Blanq. and Maire 4x = 44). Chromosome counts were previously performed by cytological techniques in the three studied species [49]. *Centaurea seridis* and *C. aspera* can coexist in Spanish natural-contact zones to produce triploid hybrids (*C. × subdecurrens* Pau 3x = 33) [32]. Similarly, *C. gentilii* and *C. seridis* can coexist in Morocco to produce tetraploid hybrids (*C. × paucispina* (Ferriol, Merle and Garmendia) P.P. Ferrer 4x = 44) [46,49]. The existence of these taxa with different ploidy levels, geographical distributions, fertility, and mating systems allows for direct comparison to analyze their competitive advantages or disadvantages. 

In this context, we aimed to study the mating system and reproductive barriers among *Centaurea aspera* and its polyploid relatives, allotetraploid *C. seridis* (4x = 44), and autotetraploid *C. gentilii* (4x = 44), and to analyze their geographical distribution. We specifically addressed the following questions: (i) what are the geographical distribution and ploidy level of the Moroccan populations? (ii) Do the seed sets that derive from the intra-specific crosses within each taxon differ? (iii) Is hybridization between *C. aspera* and *C. gentilii* possible? (iv) Are the seed sets per capitulum that derive from intra- and inter-specific crosses different? We combined previously published information with the new data to offer an overview of the geographical distribution and reproductive behavior of the three species and their hybrids.

## 2. Results

### 2.1. Centaurea gentilii and C. seridis Geographical Distribution and Ploidy Level in Morocco

The northernmost observed locality of *Centaurea gentilii* was Zaouiat el Kourati (52 km north of Essaouira). No new populations of *C. gentilii* were found on the Atlantic northern coast (from Casablanca to Tangier) and the Mediterranean coast (from Tangier to Nador). Instead, populations of *C. seridis* were frequently found in these coastal habitats, with Essaouira being the southernmost population. 

**Figure 1:**
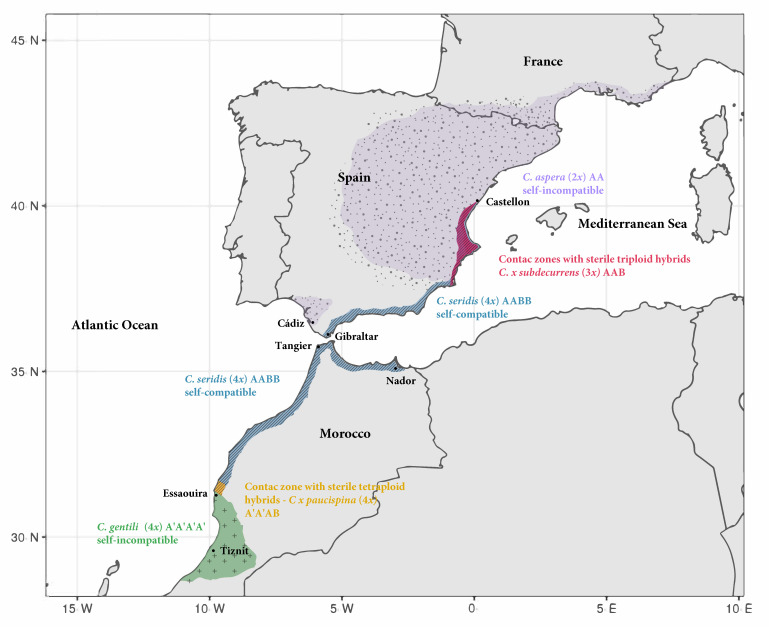
Schematic geographical distribution of *Centaurea gentilii*, *C. seridis,* and *C. aspera* with natural-contact zones with hybrids (Spain and Morocco). Spanish distribution of *C. aspera*, *C. seridis,* and triploid hybrids (*C. × subdecurrens*) was taken from previous works [32,38,49].

As all the Moroccan sampled individuals were tetraploid (4×), and in accordance with the collected data, *C. gentilii* is represented by tetraploid populations on the southern Atlantic coast of Morocco, while *C. seridis* is represented by tetraploid populations on the northern Atlantic and Mediterranean Moroccan coasts. There is a small contact zone where both species coexist north of Essaouira (Zaouiat el Kourati; Figure 1).

### 2.2. Cypselae Production in Intra-Specific Crosses

**Figure 2:**
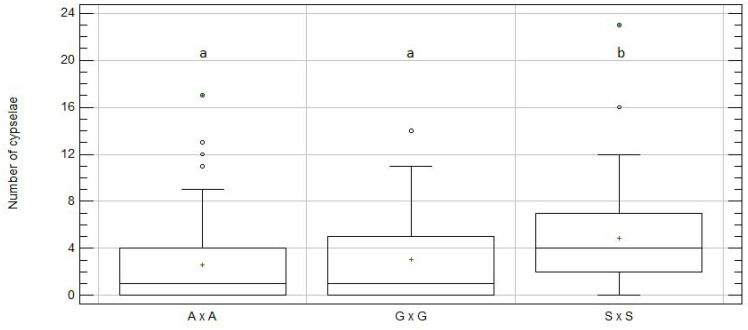
Box and whisker plot for “taxon” effect on number of cypselae per capitulum for intra-specific treatment. A × A, *C. aspera* intra-specific crosses; G × G, *C. gentilii* intra-specific crosses; S × S, *C. seridis* intra-specific crosses. Boxes show 25th and 75th percentiles. Lines in boxes denote median values. Columns with distinct letters significantly differ from one another at *p* ≤ 0.05, Degrees of freedom (Df) = 203; Kruskal–Wallis (KW) value = 12.6; *p*-value = 0.002. Zaouiat data from 2018 were excluded.

In all, 228 intra-specific crosses were performed: 48 for *C. seridis*, 98 for *C. aspera*, and 82 for *C. gentilii*. Within *C. seridis* and *C. aspera*, intra-specific crosses did not show any significant differences among populations (Figures S1 and S2, respectively). Intra-specific crosses between *Centaurea gentilii* individuals from Zaouiat performed in 2018 (zz18) resulted in an unexpected small number of cypselae, with significant differences to Zaouiat intra-specific crosses performed in 2019 (zz19) (Figure S3). All plants were sown in the greenhouse in 2018, but the Zaouiat plants grew later, and bloomed after spring in July and August when very high temperatures were recorded. This circumstance was probably the cause of the unusual infertility of this treatment in this year. Therefore, this dataset was removed from analysis, although results with or without the zz18 data did not significantly differ (Figure S4). *Centaurea seridis* produced significantly more cypselae per capitulum (4.88 ± 0.64; mean ± SE) than *C. aspera* (2.62 ± 0.35) and *C. gentilii* (3.09 ± 0.51) did (*p*-value = 0.002; Figure 2 and Table 1). Twenty-five intra-specific cypselae per species were analyzed to confirm the ploidy level and percentage of empty cypselae. All analyzed intra-specific cypselae had the same ploidy level as the progenitors did, and less than 5% empty cypselae. 

### 2.3. Intra-Specific Cypselae Production between and within Populations in C. aspera and C. gentilii

**Figure 3:**
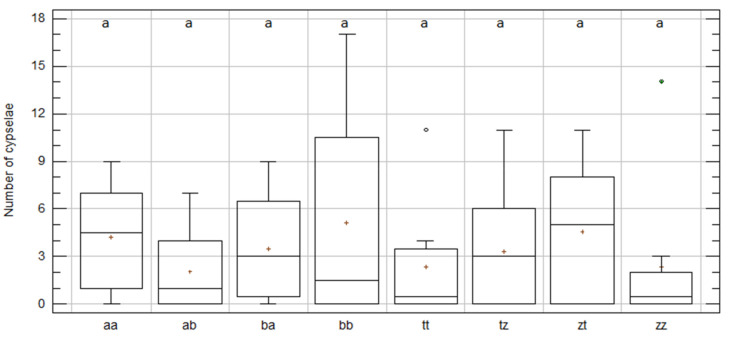
Box and whisker plots for “gamete-origin” effect on number of cypselae per capitulum for intra-specific 2019 treatment. *C. aspera* populations: aa, ovules and pollen from El Saler; ab, ovules from El Saler and pollen from Chulilla; ba, ovules from Chulilla and pollen from El Saler; bb, ovules and pollen from Chulilla; *C. gentilii* populations: tt, ovules and pollen from Tamri; tz, ovules from Tamri and pollen from Zaouiat; zt, ovules from Zaouiat and pollen from Tamri; zz, ovules and pollen from Zaouiat. Boxes show 25th and 75th percentiles. Lines in boxes show median values. Columns with same letter did not significantly differ from one another at *p* ≤ 0.05, Df = 97; KW value = 5.54; *p*-value = 0.59.

In 2019, intra-specific crosses in *C. aspera* and *C. gentilii* were specifically repeated to compare within and between populations’ seed sets. Nonsignificant differences in the number of cypselae per capitulum were found among treatments (Figure 3 and Table 2).

The crosses within the populations in *C. gentilii* (tt and zz) gave fewer cypselae per capitulum (2.38 ± 1.05) than between populations (3.97 ± 0.64), but with no statistical significance (Figure S5). The opposite happened for *C. aspera*, with more cypselae within (4.69 ± 1.32) than between populations (2.78 ± 0.5) and, once again, with no significant differences (Figure S5). Regardless of gamete origin, the *C. aspera* and *C. gentilii* intra-specific crosses performed in 2019 gave a similar number of cypselae per capitulum with no significant differences (3.42 ± 0.56 A × A vs. 3.46 ± 0.57 G × G; *p*-value = 0.94; Figure S5).

### 2.4. Inter-Specific Crosses between C. aspera and C. gentilii

Hybrid cypselae were obtained from the inter-specific treatment between *C. aspera* and *C gentilii*. Cypselae were obtained on both: *C. aspera* capitula (ovules from *C. aspera* and pollen form *C. gentilii*; A × G) and *C. gentilii* capitula (ovules from *C. gentilii* and pollen from *C. aspera*; G × A). Both species acted as ‘mothers’.

Like intra-specific treatments, the inter-specific treatments involving the *C. gentilii* population of Zaouiat, performed in 2018 (zx18), produced an extremely small number of cypselae. This denotes significant differences with the same treatment performed in 2019 (zx19) and with that performed in 2018 using the *C. gentilii* individuals from the Tamri population (tx18) (Figure S6). For this reason, the zx18 dataset was removed before running the analysis.

**Figure 4:**
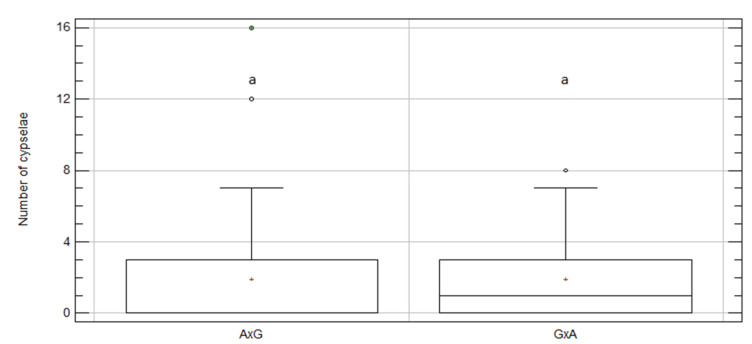
Box and whisker plot for “mother-taxon” effect on number of cypselae per capitulum for inter-specific treatment between *C. aspera* and *C. gentilii*. A × G, ovules from *C. aspera* and pollen from *C. gentilii*; G × A, ovules from *C. gentilii* and pollen from *C. aspera*. Boxes show 25th and 75th percentiles. Lines in boxes show median values. Columns with same letter did not significantly differ from one another at *p* ≤ 0.05, Df = 87; KW value = 1.18; *p*-value = 0.28.

*Centaurea aspera* and *C. gentilii* produced a similar mean number of hybrid cypselae per capitulum when the inter-specific crosses were forced, and no significant differences appeared (1.91 A × G vs. 1.93 G × A; *p*-value = 0.28; Figure 4 and Table 3), that is, hybrids were symmetrically produced. When data were separately analyzed for each year, similar results were obtained for 2018 and 2019, with no significant differences between A × G and G × A (Figure S7). 

### 2.5. Offspring Analysis

**Figure 5:**
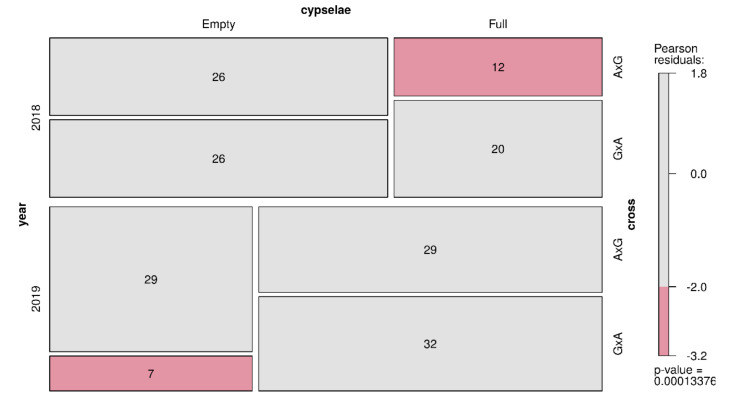
Mosaic plot of number of empty/full cypselae in each year for each crossing type. Red depicts significantly lower frequencies than expected. Numbers represent cypselae number.

We obtained 181 cypselae from inter-specific crosses, 84 in 2018 (38 A × G and 46 G × A) and 97 in 2019 (58 A × G and 39 G × A). Of these, a large number of empty (E) cypselae (48.6%) were found. The inter-specific offspring ploidy level was analyzed, and all individuals that derived from intact–full cypselae were triploid (3×) except for a single G × A tetraploid cypsela obtained in 2018. In 2018, there were fewer full A × G cypselae than expected, while there were fewer G × A empty cypselae than expected in 2019 (Figure 5). 

### 2.6. Intra- vs. Inter-Specific Treatments

**Figure 6:**
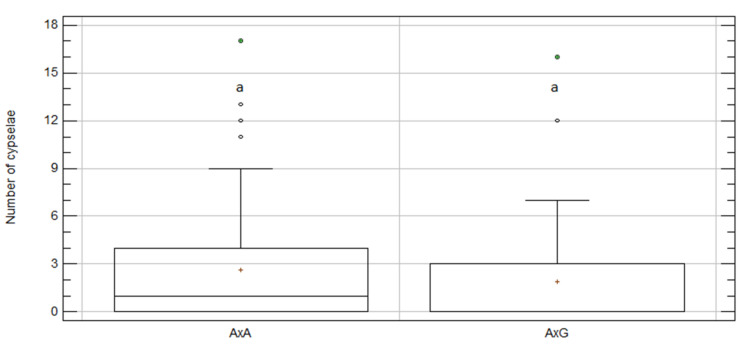
Box and whisker plot for “intra-/inter-specific-treatment” effect on number of cypselae per capitulum for *C. aspera* regardless of year. A × A, *C. aspera* intra-specific treatment; A × G, inter-specific treatment with ovules from *C. aspera* and pollen from *C. gentilii*. Boxes show 25th and 75th percentiles. Lines in boxes show median values. Columns with same letter did not significantly differ from one another at *p* ≤ 0.05; Df = 141; KW value = 3.28; *p*-value = 0.07.

Intra- and inter-specific treatments were compared within species to analyze if hybrid cypselae production was similar or not to intra-specific cypselae production. In *C. aspera*, no significant difference was found in the average number of cypselae per capitulum between intra- and inter-specific crosses (Figure 6 and Table 4). The inter-specific cross (A × G) produced fewer cypselae per capitulum (1.91 ± 0.51) than the intra-specific cross did (2.62 ± 0.35), with no significant differences (KW *p* value = 0.07). This tendency was consistent in both years (Figure S8).

**Figure 7:**
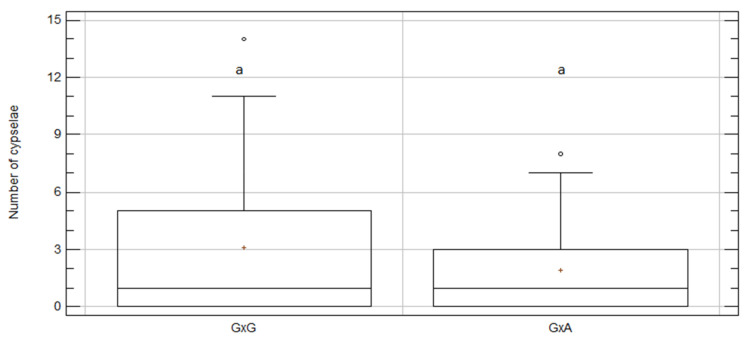
Box and whisker plot for “intra-/inter-specific-treatment” effect on number of cypselae per capitulum for *C. gentilii* regardless of year. G × G, *C. gentilii* intra-specific treatment; G × A, inter-specific treatment with ovules from *C. gentilii* and pollen from *C. aspera*. Boxes show 25th and 75th percentiles. Lines in boxes show median values. Columns with same letter did not significantly differ from one another at *p* ≤ 0.05, Df = 101; KW value = 1.06; *p*-value = 0.3.

Similarly, in *C. gentilii*, no significant difference was found in the mean number of cypselae per capitulum between the intra- and inter-specific crosses (Figure 7, Table 5). The inter-specific cross (G × A) produced fewer cypselae per capitulum (1.93 ± 0.36) than the intra-specific cross did (3.09 ± 0.51), but with no significant difference (KW *p*-value = 0.3). The same tendency was observed for both years (Figure S8).

## 3. Discussion

### 3.1. Biogeography of Centaurea aspera, C. gentilii, and C. seridis

*Centaurea gentilii* and *C. aspera* displayed clear allopatric distribution (Figure 1). Diploid *C. aspera* develops northwardly from Andalusia (S Spain), and tetraploid *C. gentilii* southwardly from Zaouiat (Essaouira, Morocco). This allopatric distribution of *C. aspera* and *C. gentilii* could have been influenced by the occurrence of the physical barrier of the Strait of Gibraltar. *C. gentilii* distribution may represent the southernmost limit of the ancestor species’ distribution.

Allotetraploid *C. seridis*, a coastal specialist that occasionally extends inland [49], occupies the intermediate area between *C. aspera* and *C. gentilii*. As a result, contact zones with both species arise: to the north of its distribution range, it forms several contact zones with *C. aspera*, in which sterile triploid hybrids (*C. × subdecurrens*) are produced [32]. In southern Morocco, a contact zone with *C. gentilii* was found with sterile tetraploid hybrids (*C. × paucispina*) [49] (Figure 1). 

We did not find *C. aspera* on the SE Mediterranean Spanish coast (form Gibraltar to Almería), but it reappeared on the SW Atlantic Spanish coast (Cádiz), where *C. seridis* was not present. This could hypothetically indicate that *C. seridis* was able to displace *C. aspera* from the southeastern Spanish coast. Both species still coexist in northern Mediterranean areas from Cartagena to Castellon. Perhaps this coexistence in the north is because *C. aspera* flows from inland populations to the coast. Something similar could have happened on the coast of Morocco between *C. seridis* and *C. gentilii*.

### 3.2. Mating System of Centaurea aspera Polyploid Complex

**Figure 8:**
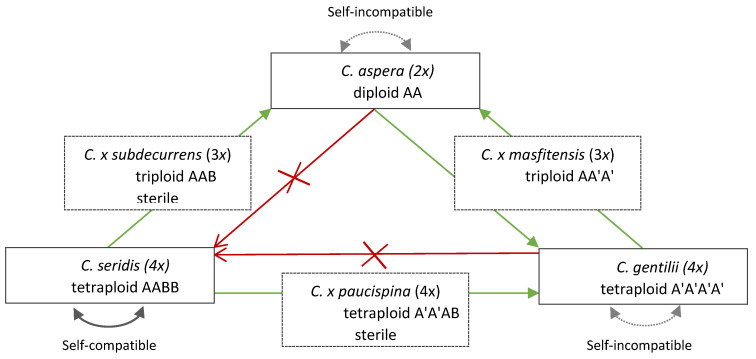
Mating-system scheme within and between three studied species (*C. aspera*, *C. seridis*, *C. gentilii*). Arrows indicate direction of cross father > mother. Green arrows denote crosses with hybrid formation. Red arrows depict blocked crosses due to pollen competition.

Diploid *C. aspera* and tetraploid *C. gentilii* are self-incompatible, while tetraploid *C. seridis* is self-compatible [32,52]. Inter-specific crosses among the three taxa are possible, but not all of them occur in both directions (Figure 8). 

*Centaurea seridis* self-compatibility forces the asymmetric formation of hybrids due to pollen competition on the stigma [32]. Therefore, *C. seridis* (4×) and *C. aspera* (2×) cross in natural contact zones with an asymmetric formation of sterile triploid hybrids (*C. × subdecurrens*), in which only *C. aspera* acts as a mother [32]. Similarly, *C. seridis* (4×) and *C. gentilii* (4×) cross in natural contact zones with an asymmetric formation of sterile tetraploid hybrids (*Centaurea × paucispina*). In this case, only *C. gentilii* acts as the mother [46,49]. 

*Centaurea aspera* and *C. gentilii* are also able to cross. The inter-specific crosses between both species resulted in the symmetrical formation of hybrids with fertile ovules and pollen from both progenitors. However, their distribution is allopatric without known natural contact zone. The triploid hybrid between these two taxa was obtained for the first time in the greenhouse of the present study, and was named *C. × masfitensis* [53]. The fertility of the new hybrid between *C. aspera* and *C. gentilii* is unknown, and new experiments are necessary to clarify this point.

### 3.3. Influence on Polyploid Establishment

Our results showed that the allotetraploid *C. seridis* produces almost twice the number of cypselae in the intra-specific crosses compared to *C. aspera* and *C. gentilii*. It is a bigger hairier plant that provides greater adaptability to coastal sandy habitats [32,38]. In the contact zones with *C. aspera*, asymmetric hybridization, along with the short distance dispersal of the hybrid cypselae, produces a differentiated microscale distribution with the sterile hybrid closer to the mother *C. aspera* [38]. All these characteristics surely favored the initial establishment and competition of *C. seridis* in sympatry with its progenitors. Selfing and high heterozygosity [47] probably enabled *C. seridis* to overcome initial bottlenecks, while phenotype differentiation, microspatial segregation, and especially the asymmetric formation of hybrids were powerful strategies to overcome the minority-cytotype-exclusion (MCE) effect described by Levin [54].

Our results also revealed that tetraploid *C. gentilii* and diploid *C. aspera* produce a similar number of cypselae in intra-specific crosses with no significant differences. *Centaurea gentilii* has a phenotype that is indistinguishable in the field from that of *C. aspera* [49]. Hybrid cypselae production was consistently lower, but without any significant differences with the intra-specific seed set. However, almost half the hybrid cypselae were empty (49%), which probably revealed postzygotic barriers. This agrees with the results observed in other Asteraceae inter-specific crosses [55].

Therefore, *C. gentilii* does not have the same advantages as *C. seridis*, which would help it to establish and compete with its diploid parental. Natural autotetraploids arise within diploid populations, and often share the ecological niche [12,13]. In sympatry, diploids and autopolyploids compete for the same biotic and abiotic resources, and the minority-cytotype-exclusion effect acts on the less abundant cytotype [54]. This has led several authors to assert that the combined challenges of MCE, meiotic abnormalities, and competition with diploids may cause most nascent polyploids to become extinct [7]. 

With an indistinguishable field phenotype, a similar seed set, self-incompatibility, and possible and symmetric inter-specific crosses, how did *C. gentilii* overcome the initial bottlenecks and escape from MCE? How did *C. gentilii* manage to establish and compete with the diploid cytotype? A substantial competitive advantage was probably required, but what was it? 

Several hypotheses were highlighted to at least partially explain the establishment of neo-auto-polyploids. The first is the recurrent formation of autopolyploids within diploid populations under environmental stress [37,56]; these nascent autopolyploids were probably of multiple origin that would increase genetic diversity and bring about a new cytotype population size [57,58]. The second would be a polyploid’s greater phenotypic plasticity that could also help it to establish, but very little evidence was provided for this. Indeed, when grown in the greenhouse, *C. gentilii* at first glance showed distinct phenotypical traits that were not observed in the field (unpublished results). Some studies already reported substantial modifications to polyploid phenotypes when grown in greenhouses [59], which could indicate greater phenotypic plasticity compared to that of their diploid progenitors. In *Knautia serpentinicola*, closely related diploids and tetraploids responded differently to key environmental factors, with the autotetraploid being more competitive than the diploid is [60].

Lastly, the hypothesis that one of the “per se advantages” of autopolyploids is the increased tolerance to inbreeding was expressed several times, but no broad consensus has been reached. Autopolyploids may effectively mask deleterious alleles better than diploids can, while allopolyploids are expected to display similar chromosomal behavior to that of diploids, and may not exhibit increased tolerance to inbreeding [61,62,63]. Very few comparative data on inbreeding depression in closely related polyploid and diploid taxa are available [25,64], but at least two studies indicated less inbreeding depression in autopolyploids in relation to in diploids [65,66]. However, there are some cases in which more inbreeding depression was observed in autopolyploids than in diploids [67].

Natural *Centaurea gentilii* populations in Morocco grow under extreme environmental conditions. Long drought periods with extreme aridity, heat waves, and heavy recurrent grazing can dramatically reduce the number of individuals. When resampling *C. gentilii* populations from 2016 to 2017, we found some populations in catastrophic condition, and most individuals had died. Under such stressful conditions, possessing more tolerance to inbreeding could facilitate the re-establishment of populations from a very small number of individuals by probably contributing to *C. gentilii* establishment. Further genetic studies into Moroccan populations would help to shed some light on the genetic diversity, population differentiation, and gene flow of *C. gentilii* in relation to past bottlenecks and inbreeding.

Although these hypotheses were discussed to explain *C. gentilii* establishment, it remains an unresolved question. Further questions, like the origin of allotetraploid *C. seridis* through secondary contact between formerly allopatric taxa, which is called the secondary-contact hypothesis [68], or the fertility or sterility of the new hybrid (*C. × masfitensis*) acting or not as a triploid bridge between *C. aspera* and *C. gentilii*, will be investigated to provide new data to help us better understand the establishment and expansion of these polyploids.

## 4. Materials and Methods

### 4.1. Geographical Distribution and Ploidy Level of Moroccan Populations

The geographical distribution, ploidy level, and genetic analyses of the Spanish populations of *C. aspera* and *C. seridis* were already reported [47,48]. Moroccan populations were surveyed for the first time in 2013. Then, eight populations of *C. gentilii* were located on the Atlantic coast of Morocco [49]. During two new expeditions (2016 and 2017), the Atlantic and Mediterranean coasts of Morocco were exhaustively resurveyed from Tiznit (southern point) to Nador (northeastern point). To determine the populations’ ploidy level, individuals were sampled during the 2016 expedition, 309 from four *C. gentilii* populations, and 96 from the *C. seridis* populations (Table 6). Thirty *C. aspera* individuals from the Spanish populations were resampled to confirm their ploidy level. The ploidy level of these individuals was determined by flow cytometry as described by Garmendia et al. (2015). 

### 4.2. Controlled Pollinations

During the 2017 expedition, cypselae were collected for forced-pollination experiments. Cypselae were sampled from two natural populations of each species (Table 1). The sampled capitula from the natural populations were stored at 4 °C for 2 months. In all cases, the sampled capitula came from 4–5 mothers under open-pollination conditions. One hundred cypselae were randomly extracted from the mixture of capitula sampled from each population and germinated. Individuals of the three species were grown in the Centro para la Investigación y la Experimentación Forestal (CIEF; Quart de Poblet, Spain) greenhouse. At least 50 plants from each species and population were grown in pots for the experiments done in January 2018.

Four controlled pollination, treatments were performed in these plants: intra-specific crosses within the three species, and inter-specific crosses between *C. aspera* and *C. gentilii*. The treated capitula were randomly selected from those available to obtain at least 30 treated capitula per treatment and taxon. Treatments were run during the flowering period, from June to August 2018. Pollinations were performed with the newly open capitula bagged in semipermeable nylon bags prior to anthesis. Upon anthesis, capitula were brushed gently against one another once a day on 2 consecutive days. During the cross-pollinations, each treated capitulum received pollen from the one-paired capitula from a different individual. The flowers of the treated capitula were not emasculated.

Due to plant management, the Zaouiat individuals grew late and bloomed outside the 2018 season, which was why the inter-specific crosses between *C. aspera* and *C. gentilii* from Zaouiat were repeated in 2019. Additionally, in 2019, the intra-specific crosses in *C. aspera* and *C. gentilii* (both allogamous) were specifically repeated to compare the seed sets obtained using individuals from the same population or from different populations within the taxon. For *C. aspera*, four pollination treatments were performed: (ss), ovules and pollen from El Saler; (sc), ovules from El Saler and pollen from Chulilla; (cs), ovules from Chulilla and pollen from El Saler; (cc), ovules and pollen from Chulilla. For *C. gentilii*, four cross-treatments were also performed: (tt), ovules and pollen from Tamri; (tz), ovules from Tamri and pollen from Zaouiat; (zt), ovules from Zaouiat and pollen from Tamri; (zz), ovules and pollen from Zaouiat.

### 4.3. Progeny Analysis 

After pollinations, capitula were rebagged for 6 weeks until fruit set. For each treatment, total cypselae per capitulum were counted. Cypselae were disinfected with 0.5% NaClO solution for 20 min, washed 3 times for 5 min in distilled water, and hydrated on parafilm-closed Petri dishes for 24 h at 20 °C. Subsequently, each cypsela was cut at 2/3 from the epicotyl, and the pericarp was removed. Both empty (without embryo) and intact (with fully developed embryos) cypselae were counted. In the intact cypselae, the 1/3 distal cotyledonary tissue was used to determine the ploidy level of each embryo by flow cytometry, as described by Garmendia et al. (2015). Each sample consisted of a small piece of leaf (0.5 cm^2^) collected from the plant, to be analyzed together with a similar leaf piece taken from a diploid control plant. Samples were chopped together using a razor blade in a nucleus isolation solution (High-Resolution DNA Kit Type P, solution A; Sysmex Partec, Munster, Germany). Nuclei were filtered through a 30 µm nylon filter and stained with a 4,6-diamine-2-phenylindol (DAPI) solution (High-Resolution DNA Kit Type P, solution B; Partec). After a 5 min incubation period, stained samples were run in a CyFlow Ploidy Analyzer (Partec) flow cytometer equipped with optical parameters for the detection of DAPI fluorescence. DNA fluorochrome DAPI was excited with UV–LED at 365 nm. Histograms were analyzed with CyView software (Sysmex Partec, Munster, Germany), which determines sample peak position, coefficient of variation (CV), arithmetic mean, and median. The rest of the embryo (2/3) was placed on wet Petri dishes at room temperature and in natural light so they could germinate.

### 4.4. Statistical Analyses

The average, standard error, skewness, and kurtosis of the number of cypselae per capitulum were assessed for each pollination treatment and taxon. The normality of residuals and homogeneity of variances were checked by a Shapiro–Wilk test and Levene test, respectively. Due to the lack of normality for the *C. aspera* and *C. gentilii* residuals, nonparametric methods were selected to compare medians: the median number of cypselae was compared among treatments and repetitions by the Kruskal–Wallis rank sum [69] and post hoc Dunn’s [70] tests. Pearson residuals were used to highlight the significant differences between observed/expected frequencies of full/empty cypselae. All statistical analyses, tables, and figures were constructed using Stat graphics XVII-X64 and R language [71] with RStudio [72]. 

## 5. Conclusions

*Centaurea aspera* and *C. gentilii* showed clear allopatric distribution, whereas *C. seridis* occupies the intermediate area between both species. All three species can hybridize, but crosses only naturally occur in contact zones with *C. seridis*. Allotetraploid *C. seridis* produces more cypselae, is bigger and autogamous, and selfing forces the asymmetric formation of sterile hybrids with both *C. aspera* and *C. gentilii*. These characteristics would help *C. seridis* to avoid the minority-cytotype-exclusion effect and to become established. Tetraploid *C. gentilii* produces the same number of cypselae per capitulum as the diploid does, has an indistinguishable field phenotype, is allogamous, and symmetrically produces hybrids with *C. aspera*. Therefore, *C. gentilii* does not have the same competitive advantages as those of *C. seridis*. There is no clear evolutionary advantage of *C. gentilii* over *C. aspera*. How did *C. gentilii* overcome the minority-cytotype-exclusion effect? Is there any per se evolutionary advantage of the autopolyploid? It is still unclear. The recurrent production of neopolyploids in stress environments and more tolerance to inbreeding were highlighted to at least partially explain the establishment of these polyploids.

## Figures and Tables

**Table 1:** Number of cypselae obtained per capitulum in intra-specific treatment per taxon.

Taxon	N	Mean	SE	KW	Skew	Kurtosis	Cypselae_sum
A × A	98	2.62	0.35	a	6.59	5.50	257
G × G	58	3.09	0.51	a	3.59	0.33	179
S × S	48	4.88	0.64	b	4.94	7.01	234
Total	204	3.28	0.28	-	9.10	9.31	670

A × A, *C. aspera* intra-specific crosses; G × G, *C. gentilii* intra-specific crosses; S × S, *C. seridis* intra-specific crosses; N, number of treated capitula; SE, standard error; KW, Kruskal–Wallis test for effect of groups on mean number of cypselae *p*-value = 0.00183931 (Df = 203; KW-value = 12.5967). Treatments with distinct letters significantly differed from one another at *p* ≤ 0.05; Cypselae_sum, total number of cypselae obtained per treatment. Zaouiat data from 2018 were excluded.

**Table 2:** Number of cypselae obtained per capitulum in *C. aspera* and *C. gentilii* for intra-specific 2019 treatment per gamete origin.

Location	Sp.	N	Mean	SE	KW	Skew	Kurtosis	Cypselae_sum
aa	*C. aspera*	8	4.25	1.28	a	−0.03	−1.23	34
ab	*C. aspera*	16	2.06	0.59	a	1.52	−0.36	41
ba	*C. aspera*	16	3.50	0.79	a	0.60	−1.11	33
bb	*C. aspera*	8	5.13	2.39	a	1.16	−0.33	56
tt	*C. gentilii*	8	2.38	1.35	a	2.34	2.47	19
tz	*C. gentilii*	17	3.35	0.84	a	1.49	−0.19	19
zt	*C. gentilii*	17	4.59	1.03	a	0.42	−1.34	57
zz	*C. gentilii*	8	2.38	1.70	a	2.99	3.98	78
Total		98	3.44	0.40	-	4.57	1.39	337

*C. aspera* populations: aa, ovules and pollen from El Saler; ab, ovules from El Saler and pollen from Chulilla; ba, ovules from Chulilla and pollen from El Saler; bb, ovules and pollen from Chulilla; *C. gentilii* populations: tt, ovules and pollen from Tamri; tz, ovules from Tamri and pollen from Zaouiat; zt, ovules from Zaouiat and pollen from Tamri; zz, ovules and pollen from Zaouiat. N, number of treated capitula; SE, standard error; KW, Kruskal–Wallis test for the effect of groups on the mean number of cypselae *p*-value = 0.593788 (Df = 97; KW value = 5.54478). The treatments with the same letter do not significantly differ from one another at *p* ≤ 0.05; Cypselae_sum, total number of cypselae obtained per treatment.

**Table 3:** Number of hybrid cypselae per capitulum for inter-specific treatment between *C. aspera* and *C. gentilii* for mother taxa.

Mother Taxa	N	Mean	SE	KW	Skew	Kurtosis	Cypselae_sum
A × G	44	1.91	0.51	a	6.67	9.41	84
G × A	44	1.93	0.36	a	2.89	0.10	85
Total	88	1.92	0.31	-	8.31	12.01	169

A × G, ovules from *C. aspera* and pollen from *C. gentilii*; G × A, ovules from *C. gentilii* and pollen from *C. aspera*; N, number of treated capitula; SE, standard error; KW, Kruskal–Wallis test for the effect of groups on the mean number of cypselae *p*-value = 0.2781 (Df = 87; KW value = 1.17634). Treatments with same letter did not significantly differ from one another at *p* ≤ 0.05; Cypselae_sum, total number of cypselae obtained per treatment.

**Table 4:** Average number of cypselae per capitulum for intra- and inter-specific *C. aspera* crosses per treatment.

Treatment	N	Mean	SE	KW	Skew	Kurtosis	Cypselae_sum
A × A	98	2.62	0.35	a	6.59	5.50	257
A × G	44	1.91	0.51	a	6.67	9.41	84
Total	142	2.40	0.29	-	8.92	8.60	341

A × A*, C. aspera* intra-specific treatment; A × G, inter-specific treatment with ovules from *C. aspera* and pollen from *C. gentilii*; N, number of treated capitula; SE, standard error; KW, Kruskal–Wallis test for effect of groups on mean number of cypselae *p*-value = 0.0699471 (Df = 141; KW value = 3.28417). Treatments with same letter did not significantly differ from one another at *p* ≤ 0.05; Cypselae_sum, total number of cypselae obtained per treatment.

**Table 5:** Average number of cypselae per capitulum for intra- and inter-specific *C. gentilii* crosses by treatment.

Treatment	N	Mean	SE	KW	Skew	Kurtosis	Cypselae_sum
G × G	58	3.09	0.51	a	3.59	0.33	179
G × A	44	1.93	0.36	a	2.89	0.10	85
Total	102	2.59	0.33	-	5.65	2.38	264

G × G, *C. gentilii* intra-specific treatment; G × A, inter-specific treatment with ovules from *C. gentilii* and pollen from *C. aspera*; N, number of treated capitula; SE, standard error; KW, Kruskal–Wallis test for effect of groups on mean number of cypselae, *p*-value = 0.302426 (Df = 101; KW value = 1.06346). Treatments with the same letter do not significantly differ from one another at *p* ≤ 0.05; Cypselae_sum, total number of cypselae obtained per treatment.

**Table 6:** Sampled population for ploidy-level analysis and cypselae collection.

Population	Species	Code	UTM Coordinates	No. Ind	Cypselae	Country
Takat	*C. gentilii*	TK	29 R 440900 3347409	66	no	Morocco
Sous Massa	*C. gentilii*	SM	29 R 422360 3300019	64	no	Morocco
Cap Beddouza	*C. seridis*	CB	29 S 492206 3617401	66	no	Morocco
Zaouiat Kourati	*C. gentilii*	ZA/z	29 R 439299 3508884	65	yes	Morocco
Tamri	*C. gentilii*	TM/t	29 R 422640 3403933	114	yes	Morocco
Bouznika	*C. seridis*	b	29 R 670580 3740367	15	yes	Morocco
Axdir	*C. seridis*	a	29 R 416555 3895607	15	yes	Morocco
El Saler	*C. aspera*	s	30 R 729751 4362619	15	yes	Spain
Chulilla	*C. aspera*	c	30 R 680542 4391645	15	yes	Spain

Code, first code for ploidy-level analysis/second code for controlled pollinations; No. of ind., number of individuals sampled by flow cytometry (2016 expedition); Cypselae, populations where cypselae were collected (2017 expedition).

## Supplementary Materials

The following are available online at https://www.mdpi.com/2223-7747/9/9/1142/s1. Figure S1: *Centaurea seridis* intra-specific treatment (S × S); Figure S2: *C. aspera* intra-specific treatment (A × A); Figure S3: *C. gentilii* intra-specific treatment (G × G); Figure S4: Intra-specific cypselae production among three taxa; Figure S5: Intra-specific cypselae production within and between populations of *C. aspera* and *C. gentilii*—2019 experiments; Figure S6: *C. gentilii* Zaouiat population crosses; Figure S7: Inter-specific cross between *C. aspera* and *C. gentilii* (A × G, G × A); Figure S8: Intra-specific vs. inter-specific treatments.
