# Supplementary material for: Intra- and Inter-Specific Crosses among Centaurea aspera L. (Asteraceae) Polyploid Relatives—Influences on Distribution and Polyploid Establishment
Source: Plants (Basel). 2020 Sep 3;9(9):1142. doi: 10.3390/plants9091142 (PMC7569768; doi:10.3390/plants9091142)
Supplement: Supplementary file 1 [file plants-09-01142-s001.zip › plants-887834-supplementary-proof/Fig. S1 .docx]

***Centaurea seridis* intra-specific treatment (S × S)**

**Figure 1.** Histogram for the frequency of capitula with a specific number of cypselae per capitulum for *Centaurea seridis* intra-specific treatment (S × S).

Comparison between gamete origin

a

a

**Figure 2.** Box and whisker plot for the effect of gamete origin on de number of cypselae per capitulum for *C. seridis* intra-specific treatment; ba, ovules from Bouznika (b) and pollen from Axdir (a); ab, ovules from Axdir and pollen from Bouznika. Boxes show the 25th and 75th percentiles. Lines in the boxes show the median values. Columns with the same letter do not differ significantly from each other at *p* ≤ 0.05 (HSD), Df = 47; F-value = 0.10; *p*-value = 0.7503.

**Table 1.** Number of cypselae obtained per capitulum in *C. seridis* intraspecific treatment by gamete origin.

| **Population** | **N** | **Mean** | **Se** | **HSD** | **Skew** | **Kurtosis** | **Cypselae_sum** |
| --- | --- | --- | --- | --- | --- | --- | --- |
| ba | 24 | 4.67 | 0.92 | a | 4.80 | 8.17 | 112 |
| ab | 24 | 5.08 | 0.92 | a | 1.69 | 0.83 | 122 |
| Total | 48 | 4.88 |  | - | 4.94 | 7.01 | 234 |

Note: analysis of variance values, Df = 47; F-value = 0.10; *p*-value = 0.7503; Levene's test p-value = 0.8171. ba, ovules from Bouznika (b) and pollen from Axdir (a); ab, ovules from Axdir and pollen from Bouznika; N, number of treated capitula; Se, standard error; HSD, honestly significant difference. Same letters in HSD means that there are no significant differences between the groups. Cypselae_sum, total number of cypselae obtained per treatment.

T-test Bouznika (ba) vs. Axdir (ab)

**t = −0.320121; *p*-value = 0.750326**; There is no significant difference between the means with alpha = 0.05; (F test for Standard Deviations comparison F = 1.41126 ; *p*-value = 0.414999 (there is no significant difference between standard deviations sigma1 = sigma2)).
