# Supplementary material for: Intra- and Inter-Specific Crosses among Centaurea aspera L. (Asteraceae) Polyploid Relatives—Influences on Distribution and Polyploid Establishment
Source: Plants (Basel). 2020 Sep 3;9(9):1142. doi: 10.3390/plants9091142 (PMC7569768; doi:10.3390/plants9091142)
Supplement: Supplementary file 1 [file plants-09-01142-s001.zip › plants-887834-supplementary-proof/Fig. S2 .docx]

***C. aspera* intra-specific treatment (A × A)**

**2018**

**Figure 1.** Histogram for the frequency of capitula with a specific number of cypselae per capitulum for *Centaurea aspera* A × A intraspecific 2018 treatment. Black line represents normal distribution. Data is not normally distributed (Shapiro-Wilk = 0.690667; *p*-value = 7.32081 E-13).

**2019**

**Figure 2.** Histogram for the frequency of capitula with a specific number of cypselae per capitulum for *Centaurea aspera* A × A intraspecific 2019 treatment. Black line represents normal distribution. Data is not normally distributed (W-Shapiro-Wilk = 0.828189; *p*-value = 3.3878 E-7).

Comparison among gametes origin regardless the year.

a

a

a

a

**Figure 3.** Box and whisker plot for the influence of gamete origin on the number of cypselae per capitulum regardless the year for *C. aspera* intraspecific treatment; ss, ovules and pollen from el Saler; sc, ovules from el Saler and pollen from Chulilla; cs, ovules from Chulilla and pollen from el Saler; cc, ovules and pollen from Chulilla. Boxes show the 25th and 75th percentiles. Lines in the boxes show the median values. Columns with the same letter do not differ significantly from each other at *p* ≤ 0.05, Df = 97; KW-value = 2.90; *p*-value = 0.41.

**Table 1.** Number of cypselae obtained per capitulum in *C. aspera* intraspecific treatment by gametes origin.

| Population | N | Mean | Se | KW | Skew | Kurtosis | Cypselae_sum |
| --- | --- | --- | --- | --- | --- | --- | --- |
| ss | 8 | 4.25 | 1.28 | a | −0.03 | −1.23 | 34 |
| sc | 41 | 2.12 | 0.45 | a | 4.21 | 3.18 | 87 |
| cs | 41 | 2.32 | 0.46 | a | 3.43 | 1.10 | 95 |
| cc | 8 | 5.13 | 2.39 | a | 1.16 | −0.33 | 41 |
| Total | 98 | 2.62 | 0.35 | - | 6.59 | 5.50 | 257 |

Note: ss, ovules and pollen from el Saler; sc, ovules from el Saler and pollen from Chulilla; cs, ovules from Chulilla and pollen from el Saler; cc, ovules and pollen from Chulilla; N, number of treated capitula; Se, standard error; KW, the Kruskal-Wallis test for the effect of groups on the mean number of cypselae *p*-value = 0.40762 (Df = 97; KW-value = 2.89798). Treatment with the same letter do not differ significantly from each other at *p* ≤ 0.05; Cypselae_sum, total number of cypselae obtained per treatment.

Comparison among gametes origin in 2019 experiments.

a

a

a

a

**Figure 4.** Box and whisker plot for the influence of gamete origin on the number of cypselae per capitulum for *C. aspera* intraspecific 2019 treatments; ss, ovules and pollen from el Saler; sc, ovules from el Saler and pollen from Chulilla; cs, ovules from Chulilla and pollen from el Saler; cc, ovules and pollen from Chulilla. Boxes show the 25th and 75th percentiles. Lines in the boxes show the median values; columns with the same letter do not differ significantly from each other at *p* ≤ 0.05, Df = 47; KW-value = 2.45; *p*-value = 0.48.

**Table 2.** Number of cypselae obtained per capitulum in *C. aspera* intraspecific 2019 treatments by gametes origin.

| Population | N | Mean | Se | KW | Skew | Kurtosis | Cypselae_sum |
| --- | --- | --- | --- | --- | --- | --- | --- |
| ss | 8 | 4.25 | 1.36 | a | −0.03 | −1.23 | 34 |
| sc | 16 | 2.06 | 0.96 | a | 1.52 | −0.36 | 33 |
| cs | 16 | 3.50 | 0.96 | a | 0.60 | −1.11 | 56 |
| cc | 8 | 5.13 | 1.36 | a | 1.16 | −0.33 | 41 |
| Total | 48 | 3.42 |  | - | 3.80 | 2.87 | 164 |

Note: ss, ovules and pollen from el Saler; sc, ovules from el Saler and pollen from Chulilla; cs, ovules from Chulilla and pollen from el Saler; cc, ovules and pollen from Chulilla N, number of treated capitula; Se, standard error; KW, the Kruskal-Wallis test for the effect of groups on the mean number of cypselae *p*-value = 0.483446 (Df = 47; KW-value = 2.45516). Treatment with the same letter do not differ significantly from each other at *p* ≤ 0.05; Cypselae_sum, total number of cypselae obtained per treatment.

Comparison between gametes origin in 2018 experiments.

a

a

**Figure 5.** Box and whisker plot for the influence of gamete origin on the number of cypselae per capitulum for *C. aspera* intraspecific 2018 treatments; sc, ovules from el Saler and pollen from Chulilla; cs, ovules from Chulilla and pollen from el Saler. Boxes show the 25th and 75th percentiles. Lines in the boxes show the median values. Columns with the same letter do not differ significantly from each other at *p* ≤ 0.05, Df = 49; KW-value = 0.33; *p*-value = 0.57.

**Table 3.** Number of cypselae obtained per capitulum for *C. aspera* intraspecific 2018 treatments by gametes origin.

| **Population** | **N** | **Mean** | **Se** | **KW** | **Skew** | **Kurtosis** | | **Cypselae_sum** |
| --- | --- | --- | --- | --- | --- | --- | --- | --- |
| sc | 25 | 2.16 | 0.64 | a | 3.61 | 2.84 | 54 | |
| cs | 25 | 1.56 | 0.53 | a | 5.00 | 6.74 | 39 | |
| Total | 50 | 1.86 | 0.41 | - | 5.79 | 5.39 | 93 | |

Note: sc, ovules from el Saler and pollen from Chulilla; cs, ovules from Chulilla and pollen from el Saler; N, number of treated capitula; Se, standard error; KW, the Kruskal-Wallis test for the effect of groups on the mean number of cypselae *p*-value = 0.567382 (Df = 49; KW-value = 0.327081). Treatments with the same letter do not significantly differ from each other at *p* ≤ 0.05; Cypselae_sum, total number of cypselae obtained per treatment.
