# Supplementary material for: Intra- and Inter-Specific Crosses among Centaurea aspera L. (Asteraceae) Polyploid Relatives—Influences on Distribution and Polyploid Establishment
Source: Plants (Basel). 2020 Sep 3;9(9):1142. doi: 10.3390/plants9091142 (PMC7569768; doi:10.3390/plants9091142)
Supplement: Supplementary file 1 [file plants-09-01142-s001.zip › plants-887834-supplementary-proof/Fig. S3 .docx]

***C. gentilii* intraspecific treatment (G × G)**

**2018**

**Figure 1.** Histogram for the frequency of capitula with a specific number of cypselae per capitulum for *Centaurea gentilii* G × G intraspecific 2018 treatment. Black line represents normal distribution. Data is not normally distributed (Shapiro-Wilk = 0.283072; p-value = 1.73195E-14).

**2019**

**Figure 2.** Histogram for the frequency of capitula with a specific number of cypselae per capitulum for *Centaurea gentilii* G × G intraspecific 2019 treatment. Black line represents normal distribution. Data is not normally distributed (Shapiro-Wilk = 0.812936; *p*-value = 3.68416E-8).

Comparison between years in Zaouiat × Zaouiat crosses (zz).

b

a

**Figure 3.** Box and whisker plot for the effect of years on the number of cypselae per capitulum for *C. gentilii* intraspecific Zaouiat × Zaouiat treatment. Boxes show the 25th and 75th percentiles. Lines in the boxes show the median values. Columns with different letter significantly differ from each other at *p* ≤ 0.05, Df = 31; KW-value = 8.77; *p*-value = 0.003.

**Table 1.** Number of cypselae obtained per capitulum for *C. gentilii* intraspecific Zaouiat x Zaouiat treatment by year.

| Year | N | Mean | Se | KW | Skew | Kurtosis | Cypselae_sum |
| --- | --- | --- | --- | --- | --- | --- | --- |
| 2018 | 24 | 0.54 | 0.54 | a | 9.80 | 24.00 | 13 |
| 2019 | 8 | 2.38 | 1.70 | b | 2.99 | 3.98 | 19 |
| Total | 32 | 1.00 | 0.59 | - | 8.40 | 14.29 | 32 |

Note: N, number of treated capitula; Se, standard error; KW, the Kruskal-Wallis test for the effect of groups on the mean number of cypselae *p*-value = 0.0030639 (Df = 31; KW-value = 8.7684). Treatments with different letter significantly differ from each other at *p* ≤ 0.05; Cypselae_sum, total number of cypselae obtained per treatment.

Comparison between years in Tamri × Tamri crosses (tt).

a

a

**Figure 4.** Box and whisker plot for the influence of years on the number of cypselae per capitulum for *C. gentilii* intraspecific treatments Tamri × Tamri crosses (tt). Boxes show the 25th and 75th percentiles. Lines in the boxes show the median values. Columns with the same letter do not significantly differ from each other at *p* ≤ 0.05, Df = 15; KW-value = 1.19; *p*-value = 0.27.

**Table 2.** Number of cypselae obtained per capitulum for *C. gentilii* intraspecific Tamri × Tamri treatment by year.

| Year | N | Mean | Se | KW | Skew | Kurtosis | Cypselae_sum |
| --- | --- | --- | --- | --- | --- | --- | --- |
| 2018 | 8 | 0.75 | 0.49 | a | 1.66 | 0.00 | 6 |
| 2019 | 8 | 2.38 | 1.35 | a | 2.34 | 2.47 | 19 |
| Total | 16 | 1.56 | 0.72 | - | 4.22 | 6.25 | 25 |

Note: N, number of treated capitula; Se, standard error; KW, the Kruskal-Wallis test for the effect of groups on the mean number of cypselae *p*-value = 0.27556 (Df = 15; KW-value = 1.18885). Treatment with the same letter do not significantly differ from each other at *p* ≤ 0.05; Cypselae_sum, total number of cypselae obtained per treatment.

Comparison between gametes origin in 2018 experiments.

a

a

**Figure 5.** Box and whisker plot for the influence of gamete origin on the number of cypselae per capitulum for *C. gentilii* intraspecific 2018 experiments. tt, ovules and pollen from Tamri; zz, ovules and pollen from Zaouiat. Boxes show the 25th and 75th percentiles. Lines in the boxes show the median values. Columns with the same letter do not significantly differ from each other at *p* ≤ 0.05, Df = 31; KW-value = 2.67; *p*-value = 0.10.

**Table 3.** Number of cypselae obtained per capitulum for *C. gentilii* intraspecific 2018 treatments by gametes origin.

| Population | N | Mean | Se | KW | Skew | Kurtosis | Cypselae_sum |
| --- | --- | --- | --- | --- | --- | --- | --- |
| tt | 8 | 0.75 | 0.49 | a | 1.66 | 0.00 | 6 |
| zz | 24 | 0.54 | 0.54 | a | 9.80 | 24.00 | 13 |
| Total | 32 | 0.59 | 0.42 | - | 11.37 | 29.61 | 19 |

Note: tt, ovules and pollen from Tamri; zz, ovules and pollen from Zaouiat; N, number of treated capitula; Se, standard error; KW, the Kruskal-Wallis test for the effect of groups on the mean number of cypselae p-value = 0.101991 (Df = 31; KW-value = 2.67407). Treatment with the same letter do not significantly differ from each other at p ≤ 0.05; Cypselae_sum, total number of cypselae obtained per treatment.

Comparison among gametes origin in 2019 experiments.

a

a

a

a

**Figure 6.** Box and whisker plot for the influence of gamete origin on the number of cypselae per capitulum for *C. gentilii* intraspecific 2019 treatments; tt, ovules and pollen from Tamri; tz, ovules from Tamri and pollen from Zaouiat; zt, ovules from Zaouiat and pollen from Tamri; zz, ovules and pollen from Zaouiat. Boxes show the 25th and 75th percentiles. Lines in the boxes show the median values; columns with the same letter do not significantly differ from each other at *p* ≤ 0.05, Df = 49; KW-value = 3.01; *p*-value = 0.39.

**Table 4.** Number of cypselae obtained per capitulum for *C. gentilii* intraspecific 2019 treatments by gametes origin.

| Population | N | Mean | Se | KW | Skew | Kurtosis | Cypselae_sum |
| --- | --- | --- | --- | --- | --- | --- | --- |
| tt | 8 | 2.375 | 1.35 | a | 2.34 | 2.47 | 19 |
| tz | 17 | 3.35294 | 0.84 | a | 1.49 | −0.19 | 57 |
| zt | 17 | 4.58824 | 1.03 | a | 0.42 | −1.34 | 78 |
| zz | 8 | 2.375 | 1.70 | a | 2.99 | 3.98 | 19 |
| Total | 50 | 3.46 | 0.57 | - | 2.82 | −0.35 | 173 |

Note: tt, ovules and pollen from Tamri; tz, ovules from Tamri and pollen from Zaouiat; zt, ovules from Zaouiat and pollen from Tamri; zz, ovules and pollen from Zaouiat; N, number of treated capitula; Se, standard error; KW, the Kruskal-Wallis test for the effect of groups on the mean number of cypselae *p*-value = 0.390093 (Df = 49; KW-value = 3.00993). Treatment with the same letter do not significantly differ from each other at *p* ≤ 0.05; Cypselae_sum, total number of cypselae obtained per treatment.

Comparison among gametes origin regardless of the year in which the experiments were performed, without zz18 data.

a

a

a

a

**Figure 7.** Box and whisker plot for the influence of gamete origin on the number of cypselae per capitulum regardless the year for *C. gentilii* intraspecific treatment without zz18 data. tt, ovules and pollen from Tamri; tz, ovules from Tamri and pollen from Zaouiat; zt, ovules from Zaouiat and pollen from Tamri; zz, ovules and pollen from Zaouiat. Boxes show the 25th and 75th percentiles. Lines in the boxes show the median values; columns with different letter significantly differ from each other at *p* ≤ 0.05, Df = 57; KW-value = 6.35; *p*-value = 0.09.

**Table 5.** Number of cypselae obtained per capitulum for *C. gentilii* intraspecific treatment by gametes origin without zz18 data.

| Population | N | Mean | Se | KW | Skew | Kurtosis | Cypselae_sum |
| --- | --- | --- | --- | --- | --- | --- | --- |
| tt | 16 | 1.56 | 0.72 | a | 4.22 | 6.25 | 25 |
| tz | 17 | 3.35 | 0.84 | a | 1.49 | -0.19 | 57 |
| zt | 17 | 4.59 | 1.03 | a | 0.42 | -1.34 | 78 |
| zz | 8 | 2.38 | 1.70 | a | 2.99 | 3.98 | 19 |
| Total | 58 | 3.09 | 0.51 | - | 3.59 | 0.33 | 179 |

Note: tt, ovules and pollen from Tamri; tz, ovules from Tamri and pollen from Zaouiat; zt, ovules from Zaouiat and pollen from Tamri; zz, ovules and pollen from Zaouiat; N, number of treated capitula; Se, standard error; KW, the Kruskal-Wallis test for the effect of groups on the mean number of cypselae *p*-value = 0.0955454 (Df = 57; KW-value = 6.35529 ). Treatments with different letter significantly differ from each other at *p* ≤ 0.05; Cypselae_sum, total number of cypselae obtained per treatment.
