# Supplementary material for: Intra- and Inter-Specific Crosses among Centaurea aspera L. (Asteraceae) Polyploid Relatives—Influences on Distribution and Polyploid Establishment
Source: Plants (Basel). 2020 Sep 3;9(9):1142. doi: 10.3390/plants9091142 (PMC7569768; doi:10.3390/plants9091142)
Supplement: Supplementary file 1 [file plants-09-01142-s001.zip › plants-887834-supplementary-proof/Fig. S4 .docx]

**Intraspecific cypselae production among the 3 taxa**

Comparison among the 3 taxa with all data

a

a

b

**Figure 1.** Box and whisker plot for the effect of ‘taxa’ on the number of cypselae per capitulum for the intraspecific treatment with all data. AxA, *C. aspera* intraspecific crosses; GxG, *C. gentilii* intraspecific crosses; SxS, *C. seridis* intraspecific crosses. Boxes show the 25th and 75th percentiles. Lines in the boxes show the median values. Columns with different letter significantly differ from each other at p ≤ 0.05, Df = 227; KW-value = 19.5; p-value = 0.00006.

**Table 1.** Number of cypselae obtained per capitulum in the intraspecific treatment by taxa.

| Location | N | Mean | Se | KW | Skew | Kurtosis | Cypselae_sum |
| --- | --- | --- | --- | --- | --- | --- | --- |
| A x A | 98 | 2.62 | 0.35 | a | 6.59 | 5.50 | 257 |
| G x G | 82 | 2.34 | 0.41 | a | 5.90 | 2.68 | 192 |
| S x S | 48 | 4.88 | 0.64 | b | 4.94 | 7.01 | 234 |
| Total | 228 | 3.00 | 0.26 | - | 10.20 | 10.37 | 683 |

Note: AxA, *C. aspera* intraspecific crosses; GxG, *C. gentilii* intraspecific crosses; SxS, *C. seridis* intraspecific crosses; N, number of treated capitula; Se, standard error; KW, the Kruskal-Wallis test for the effect of groups on the mean number of cypselae p-value = 0.0000566731 (Df = 227; KW-value = 19.5564). Treatments with different letter significantly differ from each other at p ≤ 0.05; Cypselae_sum, total number of cypselae obtained per treatment. Comparison among the 3 taxa without zz18

b

a

a

**Figure 2.** Box and whisker plot for the effect of ‘taxa’ on the number of cypselae per capitulum for the intraspecific treatment without zz18 data. AxA, *C. aspera* intraspecific crosses; GxG, *C. gentilii* intraspecific crosses; SxS, *C. seridis* intraspecific crosses. Boxes show the 25th and 75th percentiles. Lines in the boxes show the median values. Columns with different letter significantly differ from each other at p ≤ 0.05, Df = 203; KW-value = 12.6; p-value = 0.002.

**Table 2.** Number of cypselae obtained per capitulum in the intraspecific treatment by taxa without zz18 data.

| Location | N | Mean | Se | KW | Skew | Kurtosis | Cypselae_sum |
| --- | --- | --- | --- | --- | --- | --- | --- |
| A x A | 98 | 2.62 | 0.35 | a | 6.59 | 5.50 | 257 |
| G x G | 58 | 3.09 | 0.51 | a | 3.59 | 0.33 | 179 |
| S x S | 48 | 4.88 | 0.64 | b | 4.94 | 7.01 | 234 |
| Total | 204 | 3.28 | 0.28 | - | 9.10 | 9.31 | 670 |

Note: AxA, *C. aspera* intraspecific crosses; GxG, *C. gentilii* intraspecific crosses; SxS, *C. seridis* intraspecific crosses; N, number of treated capitula; Se, standard error; KW, the Kruskal-Wallis test for the effect of groups on the mean number of cypselae p-value = 0.00183931 (Df = 203; KW-value = 12.5967 ). Treatments with different letter significantly differ from each other at p ≤ 0.05; Cypselae_sum, total number of cypselae obtained per treatment.
