# Supplementary material for: Intra- and Inter-Specific Crosses among Centaurea aspera L. (Asteraceae) Polyploid Relatives—Influences on Distribution and Polyploid Establishment
Source: Plants (Basel). 2020 Sep 3;9(9):1142. doi: 10.3390/plants9091142 (PMC7569768; doi:10.3390/plants9091142)
Supplement: Supplementary file 1 [file plants-09-01142-s001.zip › plants-887834-supplementary-proof/Fig. S5 .docx]

**Intraspecific cypselae production ‘within’ and ‘between’ *C. aspera* and *C. gentilii* 2019 experiments**

a

a

a

a

**Figure 1.** Box and whisker plot for the effect of ‘taxa’ on the number of cypselae per capitulum. Within A, includes *C. aspera* crosses aa, bb; Between A, includes *C. aspera* crosses ab, ba; Within G, includes *C. gentilii* crosses tt, zz; Between G, includes *C. gentilii* crosses tz, zt. Boxes show the 25th and 75th percentiles. Lines in the boxes show the median values. Columns with the same letter do not significantly differ from each other at p ≤ 0.05, Df = 97; KW-value = 3.60; p-value = 0.31.

**Table 1.** Number of cypselae obtained per capitulum for the 2019 intraspecific treatment grouped in within and between crosses.

| Location | N | Mean | Se | KW | Skew | Kurtosis | Cypselae_sum |
| --- | --- | --- | --- | --- | --- | --- | --- |
| Within A | 16 | 4.69 | 1.32 | a | 1.65 | 0.29 | 75 |
| Between A | 32 | 2.78 | 0.50 | a | 1.58 | −1.00 | 89 |
| Within G | 16 | 2.38 | 1.05 | a | 3.55 | 3.31 | 38 |
| Between G | 34 | 3.97 | 0.67 | a | 1.28 | −1.35 | 135 |
| Total | 98 | 3.44 | 0.40 | - | 4.57 | 1.39 | 337 |

Note: Within A, includes *C. aspera* crosses aa, bb; Between A, includes *C. aspera* crosses ab, ba; Within G, includes *C. gentilii* crosses tt, zz; Between G, includes *C. gentilii* crosses tz, zt; N, number of treated capitula; Se, standard error; KW, the Kruskal-Wallis test for the effect of groups on the mean number of cypselae p-value = 0.308029 (Df = 97; KW-value = 3.59992). Treatment with the same letter do not significantly differ from each other at p ≤ 0.05; Cypselae_sum, total number of cypselae obtained per treatment.

**Intraspecific cypselae production** **between *C. aspera* and *C. gentilii* 2019 experiments**

Comparison between *C. aspera* and *C. gentilii* in 2019 experiments regardless gamete origin.

a

a

**Figure 2.** Box and whisker plot for the effect of ‘taxa’ on the number of cypselae per capitulum for 2019 intraspecific treatment. AxA, *C. aspera* intraspecific crosses; GxG, *C. gentilii* intraspecific crosses. Boxes show the 25th and 75th percentiles. Lines in the boxes show the median values. Columns with the same letter do not significantly differ from each other at p ≤ 0.05, Df = 97; KW-value = 0.006; p-value = 0.94.

**Table 2.** Number of cypselae obtained per capitulum for the 2019 intraspecific treatment by taxa.

| Location | N | Mean | Se | KW | Skew | Kurtosis | Cypselae_sum |
| --- | --- | --- | --- | --- | --- | --- | --- |
| A x A | 48 | 3.42 | 0.56 | a | 3.80 | 2.87 | 164 |
| G x G | 50 | 3.46 | 0.57 | a | 2.82 | −0.35 | 173 |
| Total | 98 | 3.44 | 0.40 | - | 4.57 | 1.39 | 337 |

Note: AxA, *C. aspera* intraspecific crosses; GxG, *C. gentilii* intraspecific crosses; N, number of treated capitula; Se, standard error; KW, the Kruskal-Wallis test for the effect of groups on the mean number of cypselae p-value = 0.939123 (Df = 97; KW-value = 0.00583265). Treatment with the same letter do not significantly differ from each other at p ≤ 0.05; Cypselae_sum, total number of cypselae obtained per treatment.
