# Supplementary material for: Intra- and Inter-Specific Crosses among Centaurea aspera L. (Asteraceae) Polyploid Relatives—Influences on Distribution and Polyploid Establishment
Source: Plants (Basel). 2020 Sep 3;9(9):1142. doi: 10.3390/plants9091142 (PMC7569768; doi:10.3390/plants9091142)
Supplement: Supplementary file 1 [file plants-09-01142-s001.zip › plants-887834-supplementary-proof/Fig. S6 .docx]

Comparison of the number of cypselae per capitulum whether *C. gentilii* population was Tamri (t) or Zaouiat (z) for the interspecific 2018 treatment.

a

b

**Figure 1.** Box and whisker plot for the effect of *C. gentilii* population on the number of cypselae per capitulum for the interspecific 2018 treatment; tx, includes interspecific crosses with *C. aspera* populations ‘s’ and ‘c’ were ovules or pollen came from Tamri (ts, tc, st, ct); zx, includes interspecific crosses with *C. aspera* populations ‘s’ and ‘c’ were ovules or pollen came from Zaouiat (zs, zc, sz, cz). Boxes show the 25th and 75th percentiles. Lines in the boxes show the median values; columns with different letter significantly differ from each other at p ≤ 0.05, Df = 109; KW-value = 18.1; p-value = 0.00002.

**Table 1.** Number of cypselae obtained per capitulum for the interspecific 2018 treatment by C. gentilii populations.

| *C. gentilii* pop | N | Mean | Se | KW | Skew | Kurtosis | Cypselae_sum |
| --- | --- | --- | --- | --- | --- | --- | --- |
| tx | 54 | 1.35 | 0.29 | a | 5.25 | 3.44 | 73 |
| zx | 56 | 0.21 | 0.12 | b | 13.30 | 28.08 | 12 |
| Total | 110 | 0.77 | 0.17 | - | 10.90 | 13.07 | 85 |

Note: tx, includes interspecific crosses with *C. aspera* populations ‘s’ and ‘c’ were ovules or pollen came from Tamri (ts, tc, st, ct); zx, includes interspecific crosses with *C. aspera* populations ‘s’ and ‘c’ were ovules or pollen came from Zaouiat (zs, zc, sz, cz); N, number of treated capitula; Se, standard error; KW, the Kruskal-Wallis test for the effect of groups on the mean number of cypselae p-value = 0.0000209212 (Df = 109; KW-value = 18.1036). Treatments with different letter significantly differ from each other at p ≤ 0.05; Cypselae_sum, total number of cypselae obtained per treatment.

Comparison of the number of hybrid cypselae per capitulum between zx18 and zx19 interspecific treatment.

a

b

**Figure 2.** Box and whisker plot for the effect of ‘year’ on the number of cypselae per capitulum for the interspecific treatment performed with *C. gentilii* Zaouiat population; zx18, includes interspecific 2018 crosses with *C. aspera* populations ‘s’ and ‘c’ were ovules or pollen came from Zaouiat (zs, zc, sz, cz); zx19, includes interspecific 2019 crosses with *C. aspera* populations ‘s’ and ‘c’ were ovules or pollen came from Zaouiat (zs, zc, sz, cz). Boxes show the 25th and 75th percentiles. Lines in the boxes show the median values; columns with different letter significantly differ from each other at p ≤ 0.05, Df = 89; KW-value = 28.8; p-value = 8.1E-8.

**Table 2.** Number of hybrid cypselae obtained per capitulum for the interspecific treatment performed with *C. gentilii* Zaouiat population by year.

| Year | N | Mean | Se | KW | Skew | Kurtosis | Cypselae_sum |
| --- | --- | --- | --- | --- | --- | --- | --- |
| zx18 | 56 | 0.21 | 0.12 | a | 13.30 | 28.08 | 12 |
| zx19 | 34 | 2.82 | 0.63 | b | 4.40 | 5.13 | 96 |
| Total | 90 | 1.20 | 0.28 | - | 12.01 | 23.29 | 108 |

Note: zx18, includes interspecific 2018 crosses with *C. aspera* populations ‘s’ and ‘c’ were ovules or pollen came from Zaouiat (zs, zc, sz, cz); zx19, includes interspecific 2019 crosses with *C. aspera* populations ‘s’ and ‘c’ were ovules or pollen came from Zaouiat (zs, zc, sz, cz); N, number of treated capitula; Se, standard error; KW, the Kruskal-Wallis test for the effect of groups on the mean number of cypselae p-value = 8.09592E-8 (Df = 89; KW-value = 28.783). Treatments with different letter significantly differ from each other at p ≤ 0.05; Cypselae_sum, total number of cypselae obtained per treatment.
