# Supplementary material for: Intra- and Inter-Specific Crosses among Centaurea aspera L. (Asteraceae) Polyploid Relatives—Influences on Distribution and Polyploid Establishment
Source: Plants (Basel). 2020 Sep 3;9(9):1142. doi: 10.3390/plants9091142 (PMC7569768; doi:10.3390/plants9091142)
Supplement: Supplementary file 1 [file plants-09-01142-s001.zip › plants-887834-supplementary-proof/Fig. S7 .docx]

**Interspecific cross between *C. aspera* and *C. gentilii* (AxG, GxA)**

Comparison of the number of hybrid cypselae per capitulum between mother taxa (gamete origin) AxG vs. GxA regardless the year.

a

a

**Figure 1.** Box and whisker plot for the effect of ‘mother taxa’ on the number of cypselae per capitulum for the interspecificc treatment between *C. aspera* and *C. gentilii*. AxG, ovules from *C. aspera* and pollen from *C. gentilii*; GxA, ovules from *C. gentilii* and pollen from *C. aspera*. Boxes show the 25th and 75th percentiles. Lines in the boxes show the median values. Columns with the same letter do not significantly differ from each other at p ≤ 0.05, Df = 87; KW-value = 1.18; p-value = 0.28.

**Table 1.** Number of hybrid cypselae per capitulum for the interspecific treatment between *C. aspera* and *C. gentilii* by mother taxa.

| Mother taxa | N | Mean | Se | KW | Skew | Kurtosis | Cypselae_sum |
| --- | --- | --- | --- | --- | --- | --- | --- |
| AxG | 44 | 1.91 | 0.51 | a | 6.67 | 9.41 | 84 |
| GxA | 44 | 1.93 | 0.36 | a | 2.89 | 0.10 | 85 |
| Total | 88 | 1.92 | 0.31 | - | 8.31 | 12.01 | 169 |

Note: AxG, ovules from *C. aspera* and pollen from *C. gentilii*; GxA, ovules from *C. gentilii* and pollen from *C. aspera*; N, number of treated capitula; Se, standard error; KW, the Kruskal-Wallis test for the effect of groups on the mean number of cypselae p-value = 0.2781 (Df = 87; KW-value = 1.17634). Treatment with the same letter do not significantly differ from each other at p ≤ 0.05; Cypselae_sum, total number of cypselae obtained per treatment.

Comparison of the number of hybrid cypselae per capitulum between mother taxa (gamete origin) AxG vs. GxA in 2018

The difference was not significant between AxG mean number of cypselae per capitulum (0.96) and GxA mean (1.74) with a KW p-value = 0.10 for the 2018 interspecific treatments

Comparison of the number of hybrid cypselae per capitulum between mother taxa (gamete origin) AxG vs. GxA in 2019

The difference was not significant between AxG mean number of cypselae per capitulum (3.41) and GxA mean (2.23) with a KW p-value = 0.80 for the 2019 interspecific treatments

Comparison of the number of hybrid cypselae per capitulum between years 2018 and 2019 regardless gamete origin

a

a

**Figure 2.** Box and whisker plot for the effect of ‘year’ on the number of cypselae per capitulum for the interspecific treatment between *C. aspera* and *C. gentilii* regardless gamete origin. Boxes show the 25th and 75th percentiles. Lines in the boxes show the median values. Columns with the same letter do not significantly differ from each other at p ≤ 0.05, Df = 87; KW-value = 3.67; p-value = 0.055.

**Table 2.** Number of hybrid cypselae per capitulum for the interspecific treatment between *C. aspera* and *C. gentilii* by year.

| Year | N | Mean | Se | KW | Skew | Kurtosis | Cypselae_sum |
| --- | --- | --- | --- | --- | --- | --- | --- |
| 2018 | 54 | 1.35 | 0.29 | a | 5.25 | 3.44 | 73 |
| 2019 | 34 | 2.82 | 0.63 | a | 4.40 | 5.13 | 96 |
| Total | 88 | 1.92 | 0.31 | - | 8.31 | 12.01 | 169 |

Note: N, number of treated capitula; Se, standard error; KW, the Kruskal-Wallis test for the effect of groups on the mean number of cypselae p-value = 0.0554565 (Df = 87; KW-value = 3.6682). Treatment with the same letter do not significantly differ from each other at p ≤ 0.05; Cypselae_sum, total number of cypselae obtained per treatment.

Comparison of the number of hybrid cypselae per capitulum between years 2018 and 2019 in AxG crosses

The difference was not significant between 2018 mean number of cypselae per capitulum (0.96) and 2019 mean (3.41) with a KW p-value = 0.055 for the AxG interspecific treatments

Comparison of the number of hybrid cypselae per capitulum between years 2018 and 2019 in GxA crosses

The difference was not significant between 2018 mean number of cypselae per capitulum (1.74) and 2019 mean (2.23) with a KW p-value = 0.46 for the GxA interspecific treatments

****Interaction: comparison among years x gamete origin

a

a

a

a

**Figure 3.** Box and whisker plot for the effect of ‘year x gamete origin’ on the number of cypselae per capitulum for the interspecific treatment between *C. aspera* and *C. gentilii*. AxG18, ovules from *C. aspera* and pollen from *C. gentilii* in 2018 treatments; GxA18, ovules from *C. gentilii* and pollen from *C. aspera* in 2018 treatments; AxG19, ovules from *C. aspera* and pollen from *C. gentilii* in 2019 treatments; GxA19, ovules from *C. gentilii* and pollen from *C. aspera* in 2019 treatments. Boxes show the 25th and 75th percentiles. Lines in the boxes show the median values. Columns with the same letter do not significantly differ from each other at p ≤ 0.05, Df = 87; KW-value = 5.78; p-value = 0.12.

**Table 3.** Number of hybrid cypselae per capitulum for the interspecific treatment between *C. aspera* and *C. gentilii* by year x gamete origin.

| Taxa x Year | N | Mean | Se | KW | Skew | Kurtosis | Cypselae_sum |
| --- | --- | --- | --- | --- | --- | --- | --- |
| AxG18 | 27 | 0.96 | 1.37 | a | 4.73 | 4.92 | 26 |
| GxA18 | 27 | 1.74 | 1.55 | a | 3.13 | 1.45 | 47 |
| AxG19 | 17 | 3.41 | 2.16 | a | 2.74 | 2.06 | 58 |
| GxA19 | 17 | 2.24 | 1.54 | a | 0.88 | −1.08 | 38 |
| Total | 88 | 1.92 | 1.71 | - | 8.31 | 12.01 | 169 |

Note: AxG18, ovules from *C. aspera* and pollen from *C. gentilii* in 2018 treatments; GxA18, ovules from *C. gentilii* and pollen from *C. aspera* in 2018 treatments; AxG19, ovules from *C. aspera* and pollen from *C. gentilii* in 2019 treatments; GxA19, ovules from *C. gentilii* and pollen from *C. aspera* in 2019 treatments; N, number of treated capitula; Se, standard error; KW, the Kruskal-Wallis test for the effect of groups on the mean number of cypselae p-value = 0,122754 (Df = 87; KW-value = 5.78116). Treatment with the same letter do not significantly differ from each other at p ≤ 0.05; Cypselae_sum, total number of cypselae obtained per treatment.
