# Supplementary material for: Intra- and Inter-Specific Crosses among Centaurea aspera L. (Asteraceae) Polyploid Relatives—Influences on Distribution and Polyploid Establishment
Source: Plants (Basel). 2020 Sep 3;9(9):1142. doi: 10.3390/plants9091142 (PMC7569768; doi:10.3390/plants9091142)
Supplement: Supplementary file 1 [file plants-09-01142-s001.zip › plants-887834-supplementary-proof/Fig. S8.docx]

**2018 *C. aspera***

Comparison of the number of cypselae per capitulum between intraspecific treatments (AxA) and interspecific treatments (AxG) in 2018.

a

a

**Figure 1.** Box and whisker plot for the effect of intra or interspecific treatment on the number of cypselae per capitulum for *C. aspera* regardless in 2018. AxA, *C. aspera* intraspecific treatment; AxG, interspecific treatment with ovules from *C. aspera* and pollen from *C. gentilii*. Boxes show the 25th and 75th percentiles. Lines in the boxes show the median values. Columns with the same letter do not significantly differ from each other at p ≤ 0.05, p-value = 0.11.

The difference was not significant between AxA18 mean number of cypselae per capitulum (1.86) and AxG18 mean (0.96) with a KW p-value = 0.11 for the 2018 treatments.

**2019 *C. aspera***

Comparison of the number of cypselae per capitulum between intraspecific treatments (AxA) and interspecific treatments (AxG) in 2019.

a

a

**Figure 2.** Box and whisker plot for the effect of intra or interspecific treatment on the number of cypselae per capitulum for *C. aspera* in 2019. AxA, *C. aspera* intraspecific treatment; AxG, interspecific treatment with ovules from *C. aspera* and pollen from *C. gentilii*. Boxes show the 25th and 75th percentiles. Lines in the boxes show the median values. Columns with the same letter do not significantly differ from each other at p ≤ 0.05, p-value = 0.56.

The difference was not significant between AxA19 mean number of cypselae per capitulum (3.42) and AxG19 mean (3.41) with a KW p-value = 0.56 for the 2019 interspecific treatments.

**2018 *C. gentilii***

Comparison of the number of cypselae per capitulum between intraspecific treatments (GxG) and interspecific treatments (GxA) in 2018.

a

a

**Figure 3.** Box and whisker plot for the effect of intra or interspecific treatment on the number of cypselae per capitulum for *C. gentilii* in 2019. GxG, *C. gentilii* intraspecific treatment; GxA, interspecific treatment with ovules from *C. gentilii* and pollen from *C. aspera*. Boxes show the 25th and 75th percentiles. Lines in the boxes show the median values. Columns with the same letter do not significantly differ from each other at p ≤ 0.05, p-value = 0.23.

The difference was not significant between GxG18 mean number of cypselae per capitulum (0.75) and GxA18 mean (1.74) with a KW p-value = 0.23 for the 2018 treatments.

**2019 *C. gentilii***

Comparison of the number of cypselae per capitulum between intraspecific treatments (GxG) and interspecific treatments (GxA) in 2019.

a

a

**Figure 4.** Box and whisker plot for the effect of intra or interspecific treatment on the number of cypselae per capitulum for *C. gentilii* in 2019. GxG, *C. gentilii* intraspecific treatment; GxA, interspecific treatment with ovules from *C. gentilii* and pollen from *C. aspera*. Boxes show the 25th and 75th percentiles. Lines in the boxes show the median values. Columns with the same letter do not significantly differ from each other at p ≤ 0.05, p-value = 0.45.

The difference was not significant between GxG19 mean number of cypselae per capitulum (3.46) and GxA19 mean (2.23) with a KW p-value = 0.45 for the 2019 treatments.
